# Selective Monitoring of Oxyanion Mixtures by a Flow System with Raman Detection

**DOI:** 10.3390/s18072196

**Published:** 2018-07-08

**Authors:** Félix Zapata, Fernando Ortega-Ojeda, Carmen García-Ruiz, Miguel González-Herráez

**Affiliations:** 1 Department of Analytical Chemistry, Physical Chemistry and Chemical Engineering, University of Alcalá, Alcalá de Henares, Madrid 28871, Spain; felix.zapata@uah.es (F.Z.); carmen.gruiz@uah.es (C.G.-R.); 2 University Institute of Research in Police Sciences (IUICP), Law Faculty, University of Alcalá, Alcalá de Henares, Madrid 28871, Spain; fernando.ortega@uah.es; 3 Department of Electronics, University of Alcalá, Polytechnic School, Alcalá de Henares, Madrid 28871, Spain

**Keywords:** flow analysis, oxyanions, Raman detection

## Abstract

Raman spectroscopy is a selective detection system scarcely applied for the flow analysis of solutions with the aim of detecting several compounds at once without a previous separation step. This work explores the potential of a portable Raman system in a flow system for the selective detection of a mixture of seven oxyanions (carbonate, sulphate, nitrate, phosphate, chlorate, perchlorate, and thiosulphate). The specific bands of these compounds (symmetric stretching Raman active vibrations of carbonate at 1068 cm^−1^, nitrate at 1049 cm^−1^, thiosulphate at 998 cm^−1^, phosphate at 989 cm^−1^, sulphate at 982 cm^−1^, perchlorate at 935 cm^−1^, and chlorate at 932 cm^−1^) enabled their simultaneous detection in mixtures. Although the oxyanions’ limit of detection (LOD) was rather poor (in the millimolar range), this extremely simple system is very useful for the single-measurement detection of most of the oxyanions in mixtures, without requiring a previous separation step. In addition, quantitative determination of the desired oxyanion can be performed by means of the corresponding calibration line. These are important advantages for controlling in-line processes in industries like those manufacturing fertilizers, pharmaceuticals, chemicals, or food, among others.

## 1. Introduction

As a vibrational technique, Raman spectroscopy is widely used for the chemical characterization of substances, either solid or liquid. In addition, Raman spectroscopy is particularly suitable for the analysis of aqueous samples because of the negligible Raman bands in water [1]. Multiple applications have been reported for Raman spectroscopy, in which a designated spot on a selected sample (chosen after sampling) is individually analysed seeking a particular analyte, which allows a positive detection. However, the applicability of Raman spectroscopy to the flow analysis of aqueous solutions with the aim of simultaneously detecting several analytes has been quite less researched. This is evidenced, for example, by the absence of Raman detectors in chromatographic analytical instrumentation. No commercial high-performance liquid chromatography (HPLC) system incorporates a selective Raman detector, whereas all HPLC systems incorporate a nonselective UV detector. The same occurs for gas chromatography, capillary electrophoresis, or ion chromatography. However, some pioneer studies demonstrated the compatibility of chromatographic techniques with flow Raman detection through homemade interfaces [2,3,4].

This work particularly applies the capability of flow Raman selective analysis on the simultaneous detection of oxyanions, which are common compounds found in many industries that work with biomolecules production, enzymatic fermentation, mining residues, water and waste treatment, etc. The static Raman detection of oxyanions such as sulphate, nitrate, perchlorate, carbonate, thiosulphate, and chlorate, either as solid salts or in solutions, has been extensively reported in the literature [5,6,7,8,9,10,11,12,13,14,15,16,17,18,19]. However, the simultaneous Raman detection of several oxyanions in flow solutions showed few advances along the years. In this respect, nitrate and perchlorate anions were successfully detected in-line using Raman spectroscopy after being separated by capillary electrophoresis [2]. Flow Raman analysis of oxyanions has also been performed to indicate the presence or absence of the oxyanion by in-line detection within the flow [20,21,22].

Thus, this work focused on setting up a simple flow system with Raman detection for the selective monitoring of oxyanion mixtures of carbonate, sulphate, nitrate, phosphate, chlorate, perchlorate, and thiosulphate and its application for analysing fertilizer solutions containing at least one of the abovementioned oxyanions.

## 2. Materials and Methods

### 2.1. Samples

Solutions of carbonate, sulphate, nitrate, chlorate, perchlorate, thiosulphate, and phosphate oxyanions were prepared from their sodium salts (sodium carbonate, sodium sulphate, sodium nitrate, sodium chlorate, sodium perchlorate, sodium thiosulphate, and sodium hydrogen-phosphate), which were purchased from Sigma-Aldrich (purity over 99%). These salts were dissolved in ultrapure water.

Individual standard solutions (1 mol/L) were prepared by dissolving the corresponding mass of each salt in 50 mL of ultrapure water. An Ohaus DV215CD analytical balance with a precision of five decimal places (0.00001 g) was used for weighting the salts. From these stock solutions, diluted solutions of 0.5 mol/L were prepared. Further, diluted solutions were prepared for determining the limit of detection (LOD) of each oxyanion.

A mixed stock solution containing the seven oxyanions mixture was prepared by mixing the same volume of each of the seven individual standard solutions previously prepared (1 mol/L) in such a way that the concentration of each oxyanion in the mixture was approximately 0.15 mol/L.

Nitrate solutions from 4.4 to 0.4 mol/L were additionally prepared for creating the calibration line, later used to quantify the amount of nitrate in the fertilizers.

Three commercial liquid fertilizers were purchased from a local shopping centre. The fertilizers were analysed with the aim of testing the suitability of the developed system as a preliminary flow quality control for liquid fertilizers. The nitrogen, phosphate, and potassium (NPK) declared compositions of the fertilizers were 8-6-6, 7-5-6, and 7-12-5, respectively.

The 8% nitrogen in the fertilizer 1 was declared to be as nitric (3.9%) and ammonia (4.1%) species. The 6% nitrogen of the fertilizer 2 was declared to be as nitric (2.9%), ammonia (2.7%), and ureic (1.4%) species. The 7% nitrogen in the fertilizer 3 was declared to be ammonia (4.5%) and ureic (2.5%). The phosphorus in all three fertilizers was declared as fully forming phosphorus pentoxide (P_2_O_5_) soluble in water. This likely means phosphoric acid: P_2_O_5_ + 3 H_2_O → 2 H_3_PO_4_.

Similarly, the total potassium was declared as being soluble potassium oxide (K_2_O), which chemically means potassium hydroxide: K_2_O + H_2_O → 2 KOH.

In order to better comprehend these elemental mass percentages, the concentration of the chemical species in molarity was calculated and summarized in Table 1.

### 2.2. Instrumentation

A portable deep cooled, highly sensitive i-Raman Pro spectrometer (B&W Tek, Newark, DE, USA) equipped with a flexible high-resolution E-grade fibre optic probe and a 785-nm laser was used for the flow Raman analysis. According to the specifications, the spectral resolution of the spectrometer was ±3 cm^−1^. Such a good resolution in a portable Raman instrument is reached with the help of a built-in cooling system in the detector (working at −55 ˚C). The instrumental acquisition parameters were optimized as follows: The laser power was varied from 20% (84 mW) to 100% (420 mW) in order to find the optimum working value. The higher the laser power, the larger the Raman intensity of the oxyanions’ bands. In addition, no remarkable fluorescence was observed at any laser power. Thus, the optimum laser power was 100% (420 mW). Similarly, the acquisition time and number of scans were optimized by balancing the signal-to-noise ratio and the analysis time. Finally, the Raman spectra were consecutively collected every 20 s by accumulating 10 scans of 1 s per scan. It should be noted that longer exposure times and higher number of scans, resulted in higher signal-to-noise ratio values. Nevertheless, it also rendered more time-spaced consecutive analysis, which is a crucial parameter in flow systems. Particularly, during the 10 s spent for collecting each spectrum, 250 μL of the flowing solution were analysed. However, depending on the desired volume to be analysed when collecting each spectrum, the exposure time and number of scans could be decreased (despite not being the optimum values in terms of Raman intensity).

### 2.3. Flow analysis methodology

**Figure 1:**
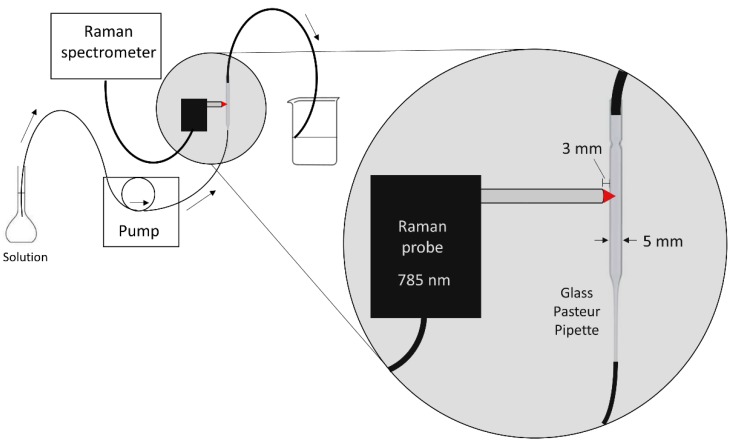
Homemade instrumental design for the flow system with Raman detection.

A volumetric flask containing the sample solution, a peristaltic pump, a glass Pasteur pipette (150 mm length, 5 mm inner diameter, soda lime glass-VWR International), and the waste beaker were connected in sequence using 0.5-mm inner diameter flexible Teflon tubes (Figure 1). The Pasteur pipette was placed vertically (inlet facing down; outlet facing up) in order to ensure a proper filling of the pipette with the solution after turning on the pump. The pump was set to 300 rpm to control the flow rate of the system, which was equivalent to 1.50 ml min^−1^. The Raman probe end was placed perpendicular to the pipette at a distance of 3 mm. This distance ensured that the spot of the probe’s lens (focal distance 5 mm), thus the laser focus, was inside the pipette, almost in its middle. The exact focal distance may have slightly varied due to the refraction of the laser when passing from the air to the glass/solution media. Positively, that variation was not significant since the Raman signal from the glass was minimal. Thereby, the Raman contribution of the pipette’s glass was minimized in such a way that it did not interfere with the solution’s Raman signal. For a few experiments, the glass pipette was replaced by a quartz standard cell cuvette (45 × 12.5 × 12.5 mm, 1 cm length, Fisherbrand, Thermo Fisher Scientific, Waltham, MA, USA) in order to check the suitability of the glass pipette compared to an ideal material (quartz).

## 3. Results and Discussion

The experimental setup and design was considerably simple and easy to implement for the intended measurements. The vertical design helped dealing with the different microconvection flow currents formed inside the pipette due to the various densities of the tested liquids. Prior horizontal or tilted trial designs of the system evidenced that denser sample liquids remained almost static inside the pipette, moved sideways forming layers, did not wash at all or did it at a painstaking pace, and/or required larger flow rates to be replaced by thinner sample liquids. On the contrary, the vertical design let the microcurrents drift up faster and rather easily in a uniform fashion. Furthermore, any occasional bubbles from the feeding line did not remain or accumulate in the measuring zone of the pipette but rose away from it towards the waste beaker. Those rare self-rising bubbles even helped cleaning the microcurrents when rinsing along the pipette. These very simple and modest attributes were paramount for such a proposed experimental device that otherwise would be plagued with drawbacks rendering a completely unpractical system.

Although the intensity of the Raman signal from the measured liquid depended quite a lot on the type of the glass pipette, the reproducibility of the readings was impressive for such a humble design. Much of the good performance of the proposed simple system was the consequence of using an external probe that allowed focusing inside the in-line sample liquid flowing along the glass pipe.

In a first step, the study examined whether there was any signal-loss when performing the flow analysis. Hence, the nitrate solution (the more representative among the solutions) was analysed several times in both static and flow regimes. No differences were observed between the two Raman spectra datasets (see supporting information). Both the static and the during-flow spectra were visually indistinguishable, showing the same nitrate band baseline and intensity. In addition, the repeatability was also the same for both gathering methods. This was evidenced by the relative standard deviation (RSD) for five consecutive measurements collected in static or during flow, which indicated a slightly better performance for the during-flow analysis compared to the static mode (0.47% and 0.39% for the static and flow measurements, respectively). This would indicate that the deviation originated in the default measurement artefacts in the spectrometer, regardless of the analysis mode (static or during-flow).

**Figure 2:**
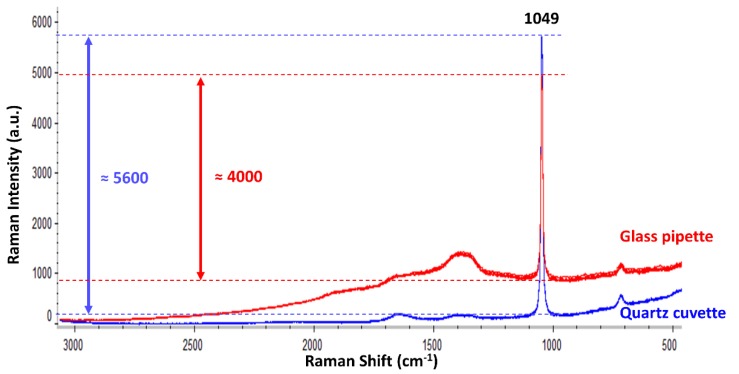
Five replicate Raman spectra for the 1M nitrate solution using either the quartz cuvette (blue) or the glass pipette (red) as flowing cells. All spectra are displayed using the same scale and no vertical offset.

In a second step, this work studied the suitability of using a glass pipette in contrast to utilising an ideal material such as a quartz cuvette. Therefore, the standard nitrate solution (one of the reference analytes) was analysed using either the glass pipette or the quartz cuvette. Figure 2 displays the Raman spectra collected when using each type of flowing cell material.

As expected, the Raman intensity of the nitrate band (1049 cm^−1^) was notably higher, and a better horizontal baseline was obtained when using the quartz cuvette. On the contrary, the glass pipette provided some fluorescence, which deviated the baseline. Nevertheless, despite such a small fluorescent signal from the glass pipette, the Raman intensity when using it was around 70% of the intensity achieved when using the quartz cuvette. Therefore, the use of a quartz cuvette is recommended because it always gave better results in all the tests. Nonetheless, in order to prove the suitability of the most simple and economic flowing system for the desired application, the following experiments are presented using the glass pipette. For the desired application (i.e., the Raman identification of different oxyanions in flowing solutions), the mandatory requirement for the flowing cell is that it must not show any interfering Raman signal within the spectral range of the oxyanion bands (1100–900 cm^−1^). Positively, neither quartz nor glass resulted in overlapping signals/fluorescence within that region.

**Figure 3:**
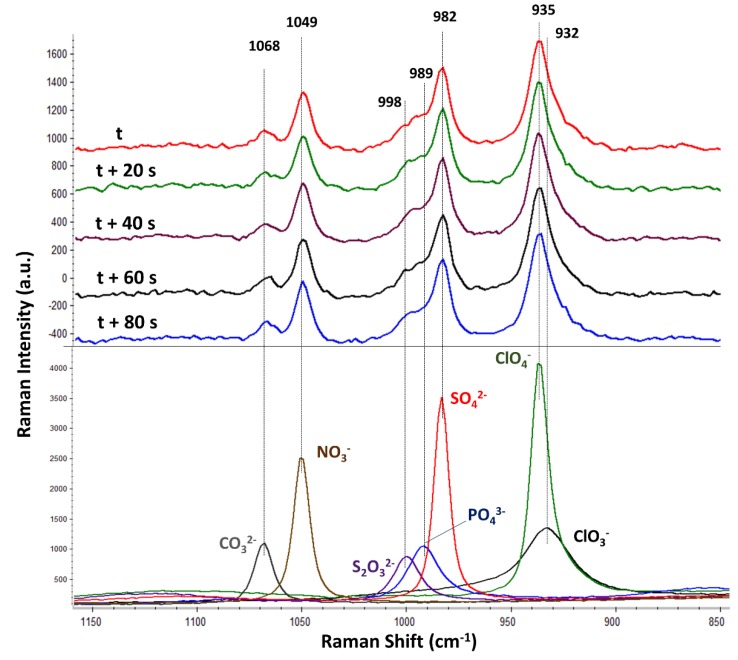
Average Raman spectra (from five replicates) of separately analysed standard solutions of carbonate, nitrate, thiosulphate, hydrogen-phosphate, sulphate, perchlorate, or chlorate, the individual concentrations of which were 0.5 mol/L each (bottom); five consecutive spectra (every 20 s) of the solution containing a mixture of the seven oxyanions, the concentration of which was 0.15 mol/L per oxyanion (top). Two scales are used: one for the standard oxyanion solutions (bottom) and another for the sample mixture containing the seven oxyanions (top). The five time-consecutive spectra of the mixture solution are vertically offset for clarity.

The Raman spectra of the seven selected oxyanions (carbonate, nitrate, thiosulphate, hydrogen-phosphate, sulphate, perchlorate, and chlorate) were initially characterized by analysing individual solutions of each oxyanion. Thereby, the knowledge about the best selective range containing the most prominent bands of each oxyanion boosted the detection of each oxyanion in the subsequent mix solution (bottom of Figure 3). In this respect, the most selective range covered from 900 to 1100 cm^−1^. This region allocates the symmetric stretching Raman active vibrations (ν1) of carbonate (1068 cm^−1^), nitrate (1049 cm^−1^), thiosulphate (998 cm^−1^), phosphate (989 cm^−1^), sulphate (982 cm^−1^), perchlorate (935 cm^−1^), and chlorate (932 cm^−1^).

It should be noted that the intensity of each oxyanion band was different in spite of all having the same molar concentration. For instance, the perchlorate band was the most intense, followed by the sulphate and nitrate bands. On the contrary, the chlorate, carbonate, phosphate, and thiosulphate bands were significantly less intense than the nitrate, sulphate, and perchlorate bands. These results clearly evidence the different Raman activity of each oxyanion vibration as follows: perchlorate > sulphate > nitrate >> chlorate ≈ carbonate ≈ phosphate ≈ thiosulphate. It should be also remarked that several species might be contributing to the same vibration due to the respective acid–base reaction of some of the oxyanions. For instance, carbonate is expected to be in three forms (carbonate, hydrogen-carbonate, and carbonic acid), whereas phosphate should be in four species (phosphate, hydrogen-phosphate, dihydrogen-phosphate, and phosphoric acid). In addition, the selective identification of each oxyanion is discussed. As previously indicated, the spectral resolution of the Raman spectrometer was ±3 cm^−1^ (according to the specifications). A simple test [23] was carried out in order to experimentally verify such a resolution (see supporting information). The test was successful since the Raman resolution enabled to resolve two proximate bands within a mixture of two solid nitrates, the spectral difference of which is known to be 7 cm^−1^ (1050 cm^−1^ for potassium nitrate and 1043 cm^−1^ for ammonium nitrate, both in solid state). Positively, the spectral shifts between the bands of carbonate (1068 cm^−1^), nitrate (1049 cm^−1^), thiosulphate (998 cm^−1^), phosphate (989 cm^−1^), sulphate (982 cm^−1^), and perchlorate/chlorate (935/932 cm^−1^) all exceed 7 cm^−1^. The exception was perchlorate/chlorate (the spectral shift of which is 3 cm^−1^) because they could not be resolved from each other. In summary, the seven oxyanions were selectively identified as carbonate, nitrate, thiosulphate, phosphate, sulphate, or a mixture of chlorate/perchlorate.

After accomplishing the flow Raman characterization of each standard oxyanion, the mixed solution containing the seven oxyanions was analysed (top of Figure 3). The figure displays five of the consecutive spectra, the measuring time lapse of which was 20 s each. The RSD among the five consecutive spectra within the 1150–850 cm^−1^ spectral range (without any preprocessing) was below 1.5% (the largest RSD was 1.5% in the most intense band belonging to perchlorate). This number showed very good results repeatability from such a simple instrumental flow design. In fact, it should be noticed that every oxyanion band was similarly intense among the five replicated spectra. Particularly, the Raman bands of nitrate, sulphate, and perchlorate dominated the spectra, while the bands of carbonate and thiosulphate were significantly less intense. The band from phosphate could not be detected. This result was not unexpected since the individual standard oxyanion solutions previously displayed the lower Raman intensity typical of the phosphate, carbonate, chlorate, and thiosulphate bands. Regarding both chlorate and perchlorate, only the vibration of perchlorate (935 cm^−1^) was observed within the maximum of the perchlorate–chlorate band.

After testing the capability of the simple flow system with Raman detection to simultaneously determine oxyanions within a flowing mixture solution (0.15 mol/L), the LOD of the proposed methodology was examined for each oxyanion. Hence, different dilutions of the 0.5 mol/L stock solutions of each oxyanion were prepared by diluting them down to a concentration of 0.002 mol/L. The LOD for each oxyanion was established as the minimum detectable concentration, that is, when the intensity of the oxyanion band exceeded at least three times the intensity of the instrumental noise (see supporting information). In brief, phosphate and thiosulphate anions showed the worst LOD (~0.05 mol/L), followed by carbonate and chlorate anions (~0.01 mol/L). Nitrate was detected down to a 0.008 mol/L value, while sulphate and perchlorate were detected down to a 0.005 mol/L value.

**Figure 4:**
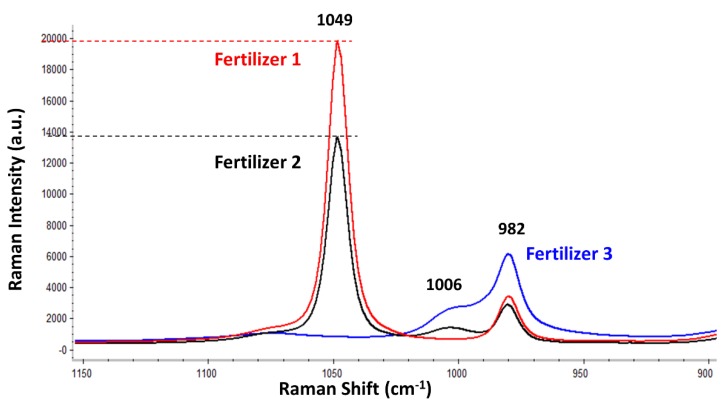
Average Raman spectra (from five replicates) from the fertilizer 1 (red), fertilizer 2 (black), and fertilizer 3 (blue). All spectra are displayed using the same scale and no vertical offset.

Finally, this work performed a practical and plain application of the developed flow analysis system for controlling the oxyanions in three different liquid fertilizers. This was done as a proof of concept, since a positive flow Raman analysis of each fertilizer would represent an advantage as a real-time, in-line quality control step within the fertilizer production line. Accordingly, Figure 4 shows the average Raman spectral signatures from the three tested fertilizers. Thus, in a real case involving those fertilizers, these Raman spectra should be obtained in every flow analysis in order to ensure that their major composition is not changing with time. Figure 4 displays the average spectra (from five replicates) of each fertilizer. It should be mentioned again that the intensity of every band in each fertilizer remained constant for the three consecutive measurements (RSD = 0.22%, 0.15%, and 0.29% for the fertilizer 1, fertilizer 2, and fertilizer 3, respectively, considering the Raman intensity of the most intense band in each fertilizer).

In brief, the fertilizers displayed up to three bands located at 1049, 1006, and 982 cm^−1^, attributed to nitrate, urea, and sulphate, respectively. Nevertheless, the relative intensity of the three bands was different for each fertilizer, which enables the discrimination and, thus, the manufacture control of each fertilizer. For instance, fertilizer 1, which had the highest concentration of nitrate, displayed the most intense nitrate band (1049 cm^−1^), followed by fertilizer 2 (the nitrate concentration of which was slightly lower, as previously summarized in Table 1). Finally, fertilizer 3, which had no nitrate in its composition according to the manufacturer, did not display the band of nitrate at 1049 cm^−1^ (as evidenced in Figure 4). Regarding the sulphate, its band (982 cm^−1^) was visible in the Raman spectra of the three fertilizers (Figure 4). Fertilizer 3 provided the most intense band of sulphate, while fertilizers 1 and 2 showed a similarly intense sulphate band. However, since the labelled NPK composition only indicated the percentage of nitrogen, phosphate, and potassium, no information about the concentration of sulphate was available in order to make any valid comparison. Finally, the band located at 1006 cm^−1^ was assigned to urea after comparing it against the Raman spectrum of the urea standard (result not shown). This band was present in the Raman spectra of fertilizers 2 and 3 but was absent in the fertilizer 1. By comparing again Table 1, it was confirmed that fertilizer 1 did not have urea, while fertilizer 3 had the most (which agrees with Figure 4).

Besides the qualitative comparison, this work tested the capability of the developed flow system to quantify the amount of nitrate in the fertilizers 1 and 2. Consequently, a calibration line was prepared for the nitrate stock solutions (4.4–0.4 mol/L). Such calibration curve allowed finding (interpolating) any intermediate Raman intensity regarding the nitrate bands of fertilizers 1 and 2. Each solution was analysed five times, and the average and standard deviation was plotted in the graphic (see supporting information). The resulting linear equation correlating the Raman intensity of the nitrate band (1049 cm^−1^) with the nitrate molar concentration (y=4648.4x+274.3) displayed a quite good coefficient of determination (*R*^2^ = 0.9991). This result clearly evidences the linear correlation between the nitrate concentration and its Raman intensity along the whole concentration range (0.4–4.4 M) tested in this work. Afterwards, the Raman intensity of the fertilizers’ nitrate band was interpolated. Hence, the nitrate concentration calculated for the fertilizer 1 was 4.05 ± 0.01 mol/L, while the concentration for the fertilizer 2 was 2.84 ± 0.01 mol/L. These values exceeded to some extent the concentrations indicated in the declared compositions of the fertilizers. However, because no concentration range was provided in their declared compositions, there is no way to know how precise the manufacture of fertilizers is. Although it was not possible to measure the exact method accuracy on those products, the precision of this approach is indeed notably high, as evidenced by the second decimal in the standard deviation.

As a summary, this simple yet effective flow Raman analysis system could be used, for example, for a very quick qualitative or quantitative check of the normal or abnormal concentration of nitrate, urea, and sulphate in a fertilizer’s manufacture in-line process.

## 4. Conclusions

A quite simple design was used to create a flow system with Raman detection for the selective monitoring of seven different oxyanions (carbonate, nitrate, thiosulphate, hydrogen-phosphate, sulphate, perchlorate, and chlorate) by means of their characteristic Raman active symmetric stretching vibrations located within the 900–1100 cm^−1^ spectral range. A glass Pasteur pipette was used as a cell flow after checking that the glass vs. quartz signal loss in the Raman intensity was not decisive.

Although the oxyanions’ LOD was rather poor (in the millimolar range), the system was very useful for the simultaneous detection of most of the oxyanions in mixtures without requiring a previous separation step. This is an important advantage for controlling in-line processes in industries like those manufacturing fertilizers, pharmaceuticals, chemicals, or food, among others. These manufacturing processes comprise some critical phases including raw materials entrance and balance, in-reactor dosing and transformation processes, waste separation and disposal, final products discharge and use, etc. Many of those production stages require a constant control usually performed manually or semimanually, with most of the steps needing rather time-, personnel-, and resource-consuming analyses.

Particularly, the presented simple flow system was preliminarily tested to control the major nitrogen composition of three different liquid fertilizers. The presence/absence attribute of the characteristic bands of each component, their respective concentration (quantitatively determined), or even their respective relative intensity were useful to test how the major composition of the fertilizer remained constant within a range of thresholds values. This simple system is being improved considering the benefits of resonant materials, which would boost the detection capabilities of such a humble device.

## Figures and Tables

**Table 1:** Molar concentration (mol/L) of the NPK components in the tested fertilizers, according to the declared compositions. No information about the non-NPK components was provided by the manufacturer.

	NO_3_^−^ (M)	NH_3_/NH_4_^+^ (M)	Urea (CO(NH_2_)_2_) (M)	H_3_PO_4_/H_2_PO_4_^−^/HPO_4_^2−^/PO_4_^3−^ (M)	K^+^ (M)
Fert1	3.4	3.4	0	2.3	1.8
Fert2	2.5	2.2	0.6	2.0	1.8
Fert3	0	4.0	1.1	4.6	1.6

## Supplementary Materials

The Supplementary Materials are available online at http://www.mdpi.com/1424-8220/18/7/2196/s1.
