# Supplementary material for: Selective Monitoring of Oxyanion Mixtures by a Flow System with Raman Detection
Source: Sensors (Basel). 2018 Jul 8;18(7):2196. doi: 10.3390/s18072196 (PMC6069446; doi:10.3390/s18072196)
Supplement: Supplementary file 1 [file sensors-18-02196-s001.pdf]

SUPPORTING INFORMATION

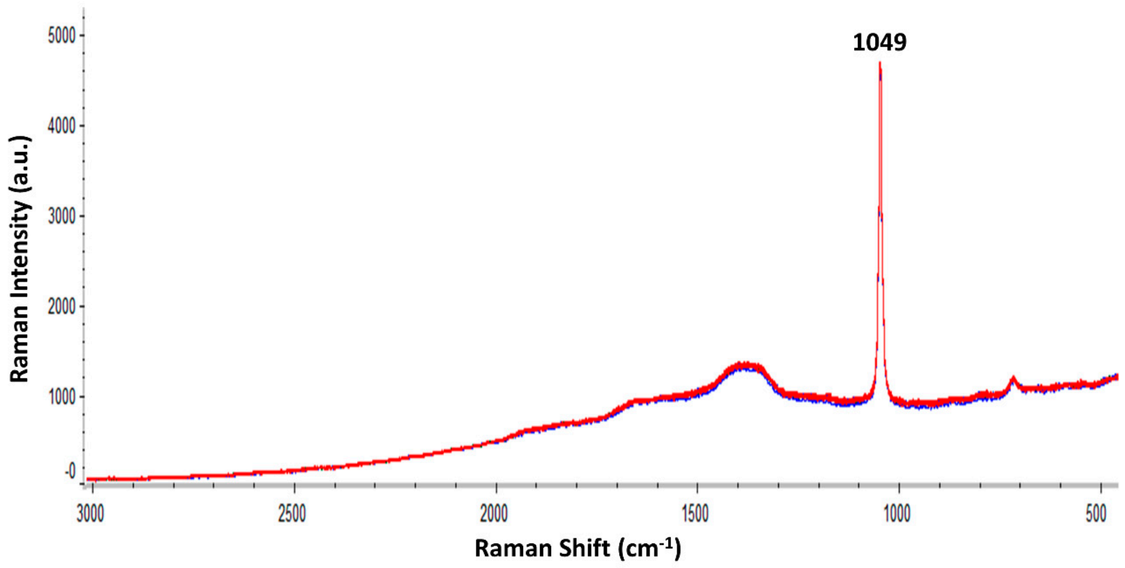

Figure S1. Five-replicate Raman spectra for the 1M nitrate solution collected either in static (red) or in during-flow (blue) modes. All spectra are displayed using the same scale and no vertical offset.

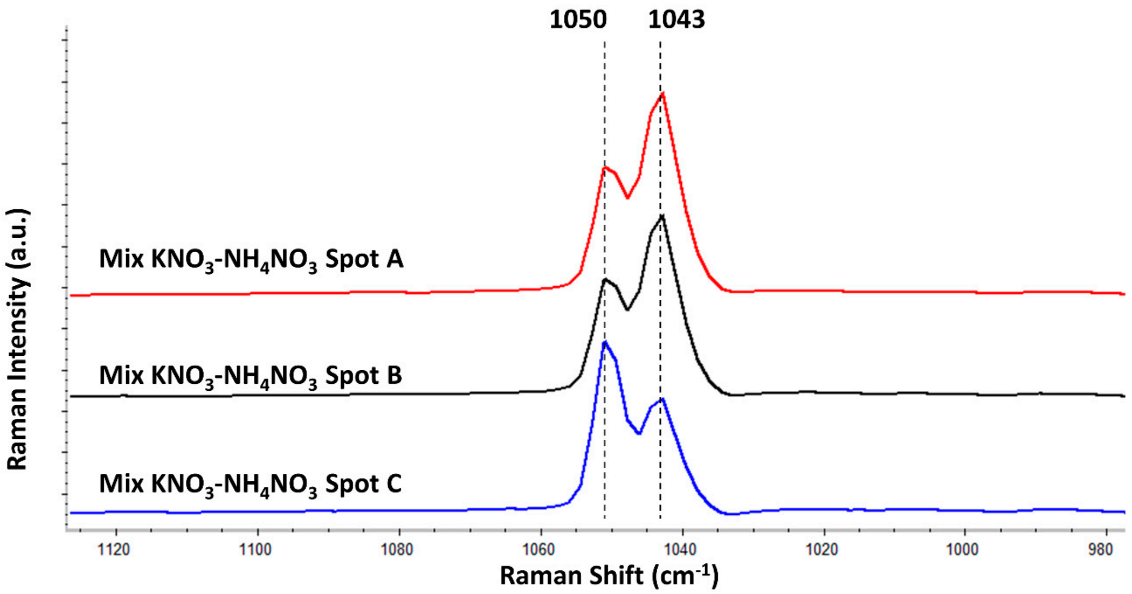

Figure S2. Test for experimentally checking the spectral resolution of the Raman spectrometer. The spectra were collected from different spots in a potassium and ammonium nitrate mixture. The bands of potassium nitrate (1050 cm^{-1}) and ammonium nitrate (1043 cm^{-1}) were resolved. All the spectra are displayed using the same scale, but they are vertically offset for clarity.

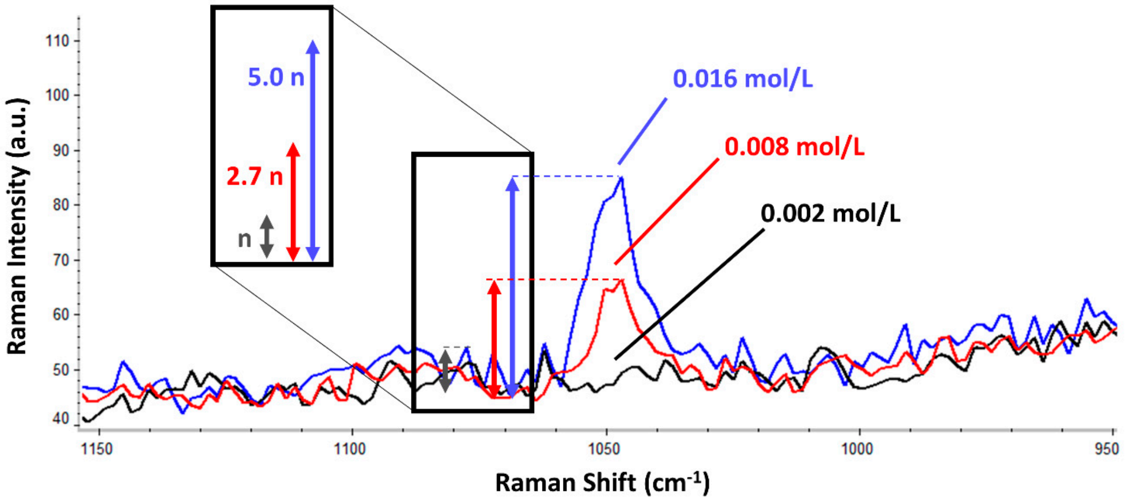

Figure S3. LOD estimation for nitrate, according to its most intense Raman band (1049 cm^{-1}). As evidenced, the Raman intensity for the 0.016M solution exceeded three times the signal-to-noise ratio (5.0), whereas the Raman intensity for the 0.008M solution was almost three times the signal-to-noise ratio (2.7). No nitrate band was perceptible for the 0.002 M concentration. All spectra are displayed using the same scale and no vertical offset.

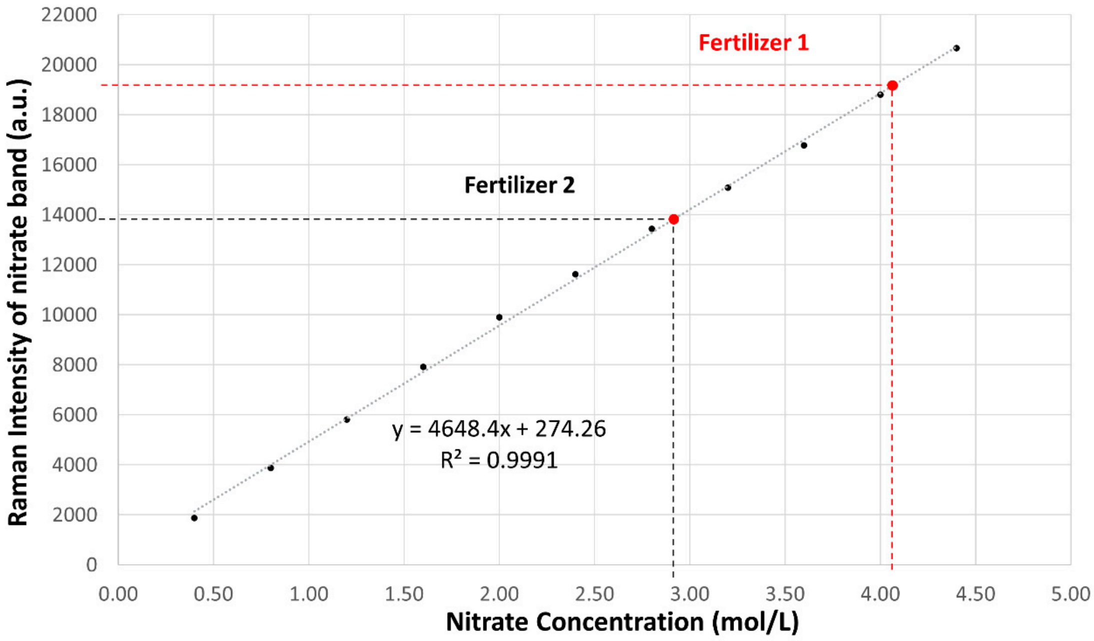

Figure S4. Quantification of the nitrate concentration in the fertilizers by interpolating the Raman intensity of the nitrate band of those fertilizers (Figure 4). The interpolation was performed on the calibration line prepared from different nitrate solutions (4.4 to 0.4 mol/L). The standard deviation vertical bars are included for each calibration measurement; however, they are not perceptible at this scale since the RSD was below 0.4 % for all the replicate measurements.
